# Refining and adapting the measurement properties of evidence-based practice measures for physiotherapy students

**DOI:** 10.1371/journal.pone.0298611

**Published:** 2024-03-07

**Authors:** Fadi M. Al Zoubi, André Bussières, Hoi Wai Chan, Kit Yat Leung, Yui Yin Ng, Ka Chun Lau, Shirley P. C. Ngai, Sharon M. H. Tsang, Arnold Y. L. Wong, Aliki Thomas

**Affiliations:** 1 Department of Rehabilitation Sciences, The Hong Kong Polytechnic University, Hong Kong, SAR, China; 2 Centre for Interdisciplinary Research in Rehabilitation of Greater Montreal, Montreal, Quebec, Canada; 3 School of Physical and Occupational Therapy, McGill University, Montreal, Quebec, Canada; 4 Département Chiropratique, Université du Québec à Trois-Rivières, Trois-Rivières, Quebec, Canada; 5 Institute of Health Sciences Education, Faculty of Medicine and Health Sciences, McGill University, Montreal, Quebec, Canada; American University of Beirut Medical Center, LEBANON

## Abstract

**Objective:**

There is a lack of reliable and valid evidence-based practice (EBP) measures for physiotherapy students. This study validated EBP-student (EBP-S) measures for physiotherapy students.

**Methods:**

EBP measures developed from previous research were cross-culturally validated for use by physiotherapy students. The adapted EBP-S consisted of six measures: use of EBP, EBP activities, EBP knowledge, self-efficacy for EBP, attitudes towards EBP, and perceptions of the teaching and assessment of EBP in the curriculum. The final version was completed by physiotherapy students (n = 335). The psychometric properties for each EBP-S measure were estimated, including construct validity using Rasch model, internal consistency reliability using person separation index (PSI), test-retest reliability using intraclass correlation coefficient (ICC), and differential item functioning (DIF).

**Results:**

Two formative measures (use of EBP and EBP activities) were only linguistically modified for use with students. A Rasch model was applied to the other four reflective measures. For knowledge, 55% (6/11) items fit the Rasch model with chi-square fit statistic (χ^2^) = 34.46, *p* = 0.08; PSI = 0.85. For self-efficacy, 89% (8/9) items fit the Rasch model with χ^2^ = 25.11, *p* = 0.80; PSI = 0.89. For attitudes, 62% (8/13) items fit the Rasch model with χ^2^ = 61.49, *p* = 0.00; PSI = 0.71. For perception of the teaching and assessment of EBP in the curriculum, 62% (8/13) items fit the Rasch model with χ^2^ = 80.99, *p* = 0.45; PSI = 0.92. perception of the teaching and assessment of EBP in the curriculum showed DIF in three items. The ICCs ranged between 0.80 and 0.98.

**Conclusions:**

The EBP-S measures were validated for physiotherapy students, including the testing of psychometric properties, which were not tested in the original studies. Further refinements should be considered for the use of the EBP-S with other groups of students or if changes are applied to the current curriculum.

## Introduction

Evidence-based practice (EBP) involves integrating high-quality evidence with clinical experience and patients’ preferences when making a clinical decision [1]. As an approach to clinical decision-making, EBP supports clinicians in the provision of high-quality care for clients [2]. The well-known 5-step EBP process is often referred to as the **5 “A’s”** of EBP: **ask** an answerable clinical question; **acquire** the best available evidence to answer the question from the literature; **appraise** the quality of the evidence, its relevance to local context, and its applicability to practice; **apply** the evidence in practice by integrating it with expertise and the patient’s views and values; and **assess** the effectiveness and efficiency of the application of the evidence and determine whether to continue to use this evidence [3].

As one of their core responsibilities and central to most professional competency standards, health care providers, including physiotherapists, are expected to use EBP in their daily practice [4, 5]. It is widely recognised that for clinicians to embrace and apply EBP, they must be exposed to what EBP is and how to operationalise it in practice during entry-level education [6, 7]. The Sicily statement on EBP recommended that all health-care education programs incorporate EBP training into their curricula [8]. While instruction on EBP for physiotherapy students is vital, a growing body of literature indicates that the measurement of EBP competencies is challenging [9–11].

While formal EBP assessments such as the Fresno Test [12] are important, they do not necessarily capture all aspects of progress or the intricacies of the learning process, which self-assessment can help capture [13]. Self-reported questionnaires can be used to assess the five EBP steps and the perceived EBP knowledge [14, 15]. In addition, self-report questionnaires have additional benefits, such as their convenient administration, cost efficiency, time efficiency, and practicality. Research conducted in various disciplines has consistently demonstrated a lack of correspondence between individuals’ self-reported skills and their actual objective performance [16]. Hence, self-assessment complements the formal evaluation processes to ensure a holistic grasp of EBP knowledge.

As a complex and highly context-specific decision-making process, EBP relies on a number of individual (e.g., knowledge, self-efficacy, attitudes, and use of EBP) and organizational (e.g., resources) factors. Assessing learners on these constructs requires the adoption of several relevant measures. In our previous work [14], we took a step towards answering a resounding call for the use of valid and reliable measures of EBP [17]. We developed and validated six EBP measures (use of EBP, EBP activities, knowledge, self-efficacy, attitudes, and resources) using Rasch measurement theory [18] in English and French among 2016–2017 graduates of the 28 physiotherapy and occupational therapy programs in Canada [14]. Given that our initial EBP measures were designed for novice clinicians [14], it is imperative to validate these measures for students in order to ascertain their efficacy, suitability, and feasibility across diverse user populations. The process of validating these measures for students enables educators and researchers to assess the efficacy of instructional methods in teaching EBP, as well as their user-friendliness and suitability for different scenarios, professional contexts, degrees of expertise, and learning preferences. This validation can also serve as a means to highlight prospective challenges, difficulties, or areas that require improvement. Those newly developed measures, which can be used to help identify the factors that influence EBP, can help inform curriculum design and revisions. Tailored curricula can better prepare physiotherapy students for their roles as evidence-based practitioners and, ultimately, lead to better patient outcomes and more effective healthcare delivery.

Given that many of these items were originally constructed for clinicians [14], the measures require additional refinements before they can be used with students. Therefore, it is necessary to conduct additional psychometric testing regarding the test-retest reliability, minimal detectable change (MDC), feasibility, floor or ceiling effects, standard error of measurement (SEM), and internal consistency for the two aforementioned measures.

The use of these measures in a different context and in other countries requires robust cross-cultural adaptation [19]. Consequently, the objectives of this study were to: (1) cross-culturally adapt EBP-student (EBP-S) measures in undergraduate and postgraduate physiotherapy programs in Hong Kong; and (2) conduct additional psychometric testing of the properties of the EBP-S measures.

## Materials and methods

This study followed the STrengthening the Reporting of OBservational studies in Epidemiology (STROBE) checklist for observational studies [20]. Ethical approval for this study was obtained from The Hong Kong Polytechnic University Institutional Review Board (reference number: HSEARS20210421001). All participants gave their informed consent in writing or electronically. The data were collected between May 3, 2021 and May 2, 2022. Our team accessed the data for purposes of data analysis between September 5, 2022 and March 3, 2023.

### Design

This study employed a two-phase process. In Phase 1, we conducted cross-cultural validation of the measures using several focus groups and pilot testing of the translated version. In Phase 2, we administered the paper or electronic self-report questionnaires to undergraduate and postgraduate physiotherapy students to evaluate the psychometric properties of the translated EBP measure. In this second phase, a cross-sectional design was used to test the construct validity of the EBP-S measures. A subset of participants from Phase 2 were then recruited to test the internal consistency and test-retest reliability of the EBP-S measures.

### EBP-S measures

This study adapted two sets of measures from earlier work [6, 14]. The first set consists of four measures (use of EBP, EBP activities, knowledge, and self-efficacy) adapted from our original work among Canadian graduates of OT and PT programs [14]. A total of 49 items represent the following constructs:

1. *Use of EBP*: The 9-items measure the actual use of EBP and reflect the 5 steps of the EBP process [3] over the past six months. A 5-point response scale was used, namely: *‘Never’*, *‘1 to 2 times’*, *‘Almost every month’*, *‘2 to 10 times a month’*, *and ‘More than 10 times a month’*.
2. *EBP Activities*: The 7-items evaluate the application of research findings in one’s practice environment, such as formally/informally sharing the recent research findings with colleagues or patients at the working/learning context. It is a 5-point scale, ranging from “never” to “daily”. The total score ranges from 0 to 140, with higher values indicating greater participation in EBP activities.
3. *Knowledge about EBP*: This is an 11-item measure that evaluates a student’s basic knowledge of EBP. Participants are required to report their understanding of research terminology, including statistical and methodological jargon. A 5-point scale of response options was used, including *‘Never heard the term’*, *‘Have heard it but do not understand’*, *‘Have some understanding’*, *‘Understand quite well’*, and *‘Understand and could explain to others’*. A greater understanding indicates more comprehensive knowledge about EBP.
4. *Self-efficacy*: The 9-items measure participants’ confidence in implementing EBP in clinical practice. On an 11-point scale ranging from 0% to 100%, participants are instructed to rate their level of confidence on their current ability to apply the 5 steps of the EBP process. A greater percentage indicates higher confidence in applying the 5 steps.

The second set of measures targets 1) attitudes towards EBP and 2) students’ perceptions of the teaching and assessment of EBP in the curriculum. The items were adapted from a previous study involving occupational therapy students at a Canadian university [6]. A total of 53 items representing the following measures were used:

5. *Attitudes towards EBP*: This 13-item measure evaluates a student’s views towards EBP. All items use a 7-point Likert scale, including *‘Strongly disagree’*, *‘Disagree’*, *‘Somewhat disagree’*, *‘Neither disagree nor agree’*, *‘Somewhat agree’*, *‘Agree’*, *and ‘Strongly agree’*.
6. *Perception of the teaching and assessment of EBP in the curriculum*: This scale consists of four subscales:
  - *Overall perception of EBP in the curriculum*: This 13-item subscale measures students’ general impressions of EBP in their curriculum, including their feelings of the program’s readiness and instructors’ abilities to deliver EBP.
  - *Perception of EBP Training in the curriculum*: This 12-item subscale evaluates students’ impressions about the strategies used to teach the EBP’s 5 steps in their curriculum.
  - *Perception of EBP evaluation in the curriculum*: This 4-item subscale evaluates students’ impressions about the assessment of EBP in their curriculum.
  - *Students’ experience of EBP in clinical placements*: This 11-item subscale evaluates students’ impressions of the learning resources and the application of EBP in the clinical context during fieldwork.

All the subscale’ items use a 7-point Likert scale, which includes ‘Strongly disagree’, ‘Disagree’, ‘Somewhat disagree’, ‘Neither disagree nor agree’, ‘Somewhat agree’, ‘Agree’, and ‘Strongly agree’.

### Phase I: Cross-cultural validation

To use the EBP measures with students, we first evaluated the influence of classroom context on the application of EBP. Upon careful examination of our original work [14], we determined that the items on resources and attitudes towards EBP were inappropriate for students because these students have not yet been employed and, as such, cannot comment on these items that relate to the workplace. Instead, we used and adapted measures pertaining to attitudes regarding EBP and perceptions of the impact of education on EBP from a prior study conducted among students in one Canadian occupational therapy program [6].

Linguistic and psychometric validation was undertaken using a cross-cultural adaptation method [19]. As English is the language of instruction in Hong Kong, these measures were translated from North American English to Hong Kong English to ensure conceptual equivalence. We initially obtained permission to adapt the two sets of measures from the developers, who are involved in this study. The translation and cross-cultural adaptation process followed the recommended guidelines [19]. The S1 File details the 6-stage process followed by this paper.

### Phase II: Testing the psychometric properties

We prepared both printed and online surveys to test the psychometric properties of the final Hong Kong English EBP-S measures following the recommended standards [21–23]. The structural validity of the EBP-S measures was tested using Rasch analysis [14].

#### Participants

The participants were students from four cohorts: two from the undergraduate physiotherapy program (BSc in their third (BSc-Y3) and fourth (BSc-Y4) year of study), and two from the postgraduate physiotherapy programs (entry level MPT in their second year (MPT-Y2) and MSc in the Manipulative Physiotherapy program in their second year (MSc-Y2). These students were chosen because they had prior EBP training and had completed at least one clinical placement. A minimum sample size of 200 students was planned because this is the recommended sample size for conducting Rasch analysis (200 and 500 participants [24–26]. For the test-retest reliability, the enrolment of 100 students was planned, which is the suggested ‘adequate’ sample size by the COSMIN checklist [22, 27]. According to the checklist, a sample size of ≥ 100 participants is considered adequate, while a sample size of 50–99 is considered good, 30–49 is considered moderate, and < 30 is considered poor [22, 27].

#### Recruitment and data collection

Participants were recruited from a research-intensive university in Hong Kong. A draw of vouchers worth up to HK$200 (~US$25) was offered to increase survey participation, a recommended method for data collection [28]. After two weeks, those interested participants to take the retest survey received a reminder email. Retest surveys were given 2–3 weeks later with two e-reminders to avoid recall bias [22] and changes in EBP knowledge that could occur with additional classroom instruction.

Participants were recruited using three invitation methods: face-to-face following in-class practical sessions with a printed survey; online with an electronic survey; and posters placed around the academic department. The study goal and survey time (10–15 minutes) were verbally, written, or electronically described. The electronic survey was created using the Qualtrics software (Qualtrics Survey2020, Utah, USA; https://www.qualtrics.com/) with a QR code.

#### Data analysis

For continuous variables, descriptive statistics were given using means (standard deviation (SD)), and for categorical variables, frequencies (percentages). All descriptive and measurement analyses were carried out using the Statistical Analysis Software (SAS) (version 9.4) [29] and the Statistical Package for the Social Sciences (SPSS v.2) [30]. All Rasch analyses were carried out using the Rasch Unidimensional Measurement Model (RUMM) Software (version 2030) [31].

#### Rasch analysis

The reliability and validity of the EBP-S measures were tested using Rasch analysis [14]. Rasch is a unidimensional model that ranks the difficulty of the items (from easy to hard) based on the ability distribution of respondents, a process known as item hierarchy. This implies that students with greater abilities (i.e., more experienced in using EBP steps) are expected to choose the higher response options, and vice versa. The items were then ordered along a continuum, from those items requiring little ability to respond at the bottom to those requiring exceptional skill at the top. Item hierarchy can be tested using an item map [32]. Masters’ partial credit Rasch polytomous model was used in our analyses, as this model is suitable for ordinal response options [33]. As previously indicated in our original study [14], two measures fit a formative model (use of EBP and EBP activities), which indicates that Rasch analysis is not needed. On the other hand, four measures align with a reflective model: attitudes, self-efficacy, knowledge, and resources. For these measures and for the perception of the teaching and assessment of EBP in the curriculum, we conducted a Rasch analysis. The following steps were followed to verify the assumptions of the Rasch model for the EBP-S measures that fit a reflective conceptual model:

*Item response thresholds*. All the items tested in the four EBP-S measures were polytomous, with at least 5 response categories. The boundaries between neighbouring categories are referred to as thresholds. These thresholds denote locations on the latent variable where the probability of any of the adjacent categories is equal [34, 35]. It is expected that these thresholds would be ordered, indicating that the transition from one score to the next is in line with the rise in the latent variable. This is referred to as monotonicity, which is one of the assumptions of the Rasch model that was checked using the item threshold parameters, a threshold map, and category probability curves. If the thresholds were disordered, the item was rescored by collapsing the adjacent response options. In certain items, the collapsing of the response options reduces the responses to become binary.

*Overall*, *person and item fit to the Rasch model*. To assess how the item/person differs from the expected responses generated by the Rasch model, we assessed the model fit using standardized fit residuals. The items and person were considered fit if the residual values were within ± 2.5 [34, 35]. Items with greater residual values than +2.5 may indicate multidimensionality, while items with lower values than -2.5 may indicate redundancy. These misfit items are now functioning the way they intended to be; therefore, they were removed from the construct. Using the summary fit residual statistics, the overall model fit was tested by a non-significant chi-square fit statistic (χ^2^) with a *p-value* > 0.05 post-Bonferroni adjustment and a non-significant F statistic for the available items. This indicates homogeneity of the items among groups with different scores [34, 35].

Unidimensionality indicates that all items within a construct should measure a single latent variable. Unidimensionality was examined using the principal component analysis of the residuals, which identified and contrasted two sets of items with opposing loadings using independent *t* tests [34, 35]. For each measure’s items to be considered unidimensional, less than 5% of *t*-test values should be significant, falling outside ± 1.96 [36].

*Structural validity*. Structural validity for a measure was tested by examining the item distribution over the hierarchical linear continuum from least to greatest difficulty. To do this, we examined the statistical and graphical distribution of the items. Ideally, the location of the item or person on the continuum should be centred at 0 and have a standard deviation (SD) of 1. We identified items with identical locations, which may imply item redundancy. In addition, we inspected any gaps throughout the estimated targeted range (−4 to +4 logits).

*Local item dependence*. Local item dependence is when ratings on one item within the same construct are reliant on scores of another item [37]. To identify dependent items, we examined the residual correlations between pair-wise items after accounting for the latent component. Any correlation greater than 0.3 was deemed dependent, affecting the measure’s reliability. To address this issue, two solutions were considered: creating a "super item" by combining the response options of the items, or retaining the item with the best phrasing [37]. The research team met to compare the degree of linguistic intricacy between two highly correlated items and opted for the one presented in a simplified language.

*Differential item functioning (DIF)*. One of the Rasch model requirements is the lack of DIF. The presence of DIF suggests the occurrence of item bias, which demonstrates that different subgroups within the same sample perform differently on the same measure item. In this study, DIF was examined across the EBP-S measures by academic cohort, prior research experience, grade point average (GPA), and sex. There was a consensus among the research team regarding these variables as the source of students’ divergent responses. An item was determined to have DIF if the *F* test employing a two-way analysis of variance was significant. In addition, we visually examined the item characteristic curve to see whether it supported the statistical finding. We explored both splitting the score and removing the item as potential solutions.

#### Internal consistency reliability

The Cronbach’s alpha (*α*) coefficient, an indicator of internal consistency reliability, was not calculated for the measures that fit the formative model, namely: use of EBP and EBP activities, as recommended [38]. For the other four measures, internal consistency reliability was examined using the Person Separation Index (PSI) using the baseline sample, which in Rasch analysis is equivalent to Cronbach’s *α*. A Cronbach’s *α* value between 0.70 and 0.95 is considered acceptable [39, 40].

#### Test–retest reliability

The relative test-retest reliability of the six EBP-S measures was tested using the two-way random effects, absolute agreement, and intraclass correlation coefficient model 2.1 (ICC (2,1) _AGREEMENT_). The ICC (2,1)_AGREEMENT_ was calculated using the formula provided by Shrout and Fleiss (1979) [41]:

$${ICC(2,1)}_{AGREEMENT}=\frac{\sigma_{x}^{2}}{\sigma_{x}^{2}{+\sigma }_{y}^{2}+\sigma_{residual}^{2}}$$

Where, $\sigma_{x}^{2}$ = variance between students, $\sigma_{y}^{2}$ = variance due to systematic differences between the two survey administrations, $\sigma_{residual}^{2}$ = residual variance.

The ICC cut-off values are: <0.50 (poor), 0.50 to 0.75 (moderate), 0.75 to 0.90 (good), and >0.90 (excellent) [42]. For individual comparisons over two-time points, an ICC of ≥ 0.90 is deemed sufficient [21, 43]. For group comparisons, an ICC of ≥ 0.70 is considered sufficient [21, 43].

#### SEM and sensitivity to change

SEM measures the inaccuracy in the scores that cannot be attributable to real changes. For each EBP-S measure, we calculated the SEM_AGREEMENT_ using the square root of the variance due to systematic differences between the two survey administrations and the residual variance. These values were derived from the ICC (2,1)_AGREEMENT_ using the following formula [44]:

$$SEM=\sqrt{\sigma_{y}^{2}+\sigma_{residual}^{2}}$$

Where ${\sigma_{T}}^{2}$ = total *variance*.

In our study, the SEM represents the standard deviation of the students’ scores over two time points.

MDC indicates the smallest change required by each student between two repeated measures to guarantee that the observed change is not attributed to measurement error [45]. The MDC values for each EBP-S measure were calculated using the SEM, with a confidence interval of 90% or 95%. MDC with a 95% confidence interval (MDC_95_) was computed using the following formula [46]: ${MDC}_{95}=1.96\times SEM\times \sqrt{2}$. The MDC with a 90% confidence interval (MDC_90_) was computed using the following formula [46]: ${MDC}_{90}=1.64\times SEM\times \sqrt{2}$.

### Feasibility and floor and ceiling effects

To assess the data feasibility, we counted the number of missing data for each EBP-S measure in the baseline data. We examined the floor and ceiling effects, which were defined as the presence of ≥15% of participants with the lowest or highest possible score [21] for two EBP-S measures: use of EBP and EBP activities. For the other four measures, we examined the floor and ceiling effects using the person-item distribution map provided by Rasch analysis.

## Results

### Phase I: Cross-cultural validation

**Fig 1:**
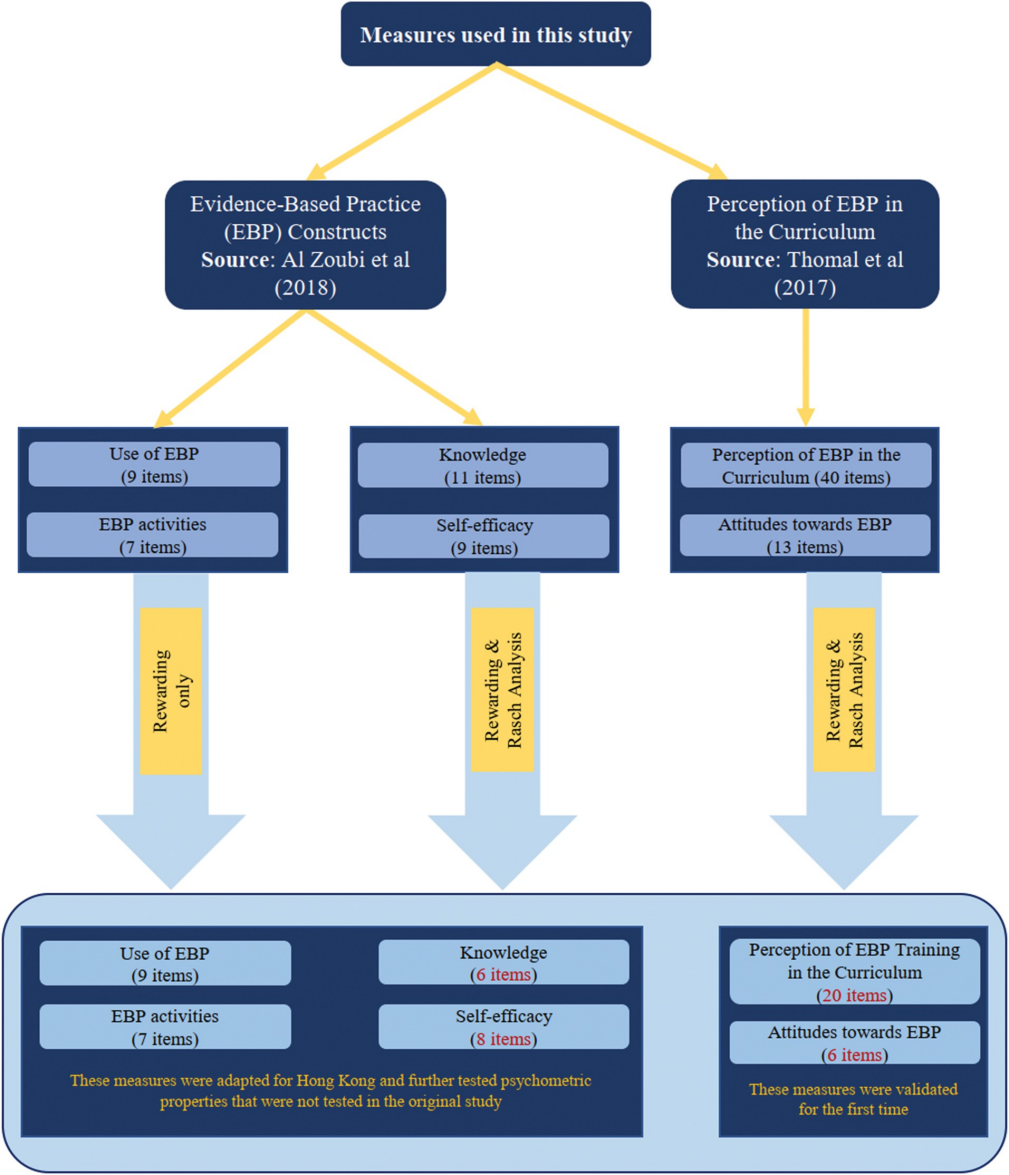
The validation process of the six EBP-S measures.

The translation Stages (I-IV) were conducted as specified in the methods. A number of uncertainties were identified, discussed, and resolved. The S1 Table details the major and minor modifications made to the items of the EBP-S measures. In Stage V, 52 participants were recruited to review the pre-final version and participate in the cognitive debriefing. The characteristics of the pilot study are summarized in the S2 Table. Cognitive debriefing interviews demonstrated that items on the six measures were understandable and relevant. On average, it took 13.5 minutes to complete the survey. Fig 1 demonstrates the validation process of the EBP-S measures.

### Phase II: Testing the psychometric properties

#### Characteristics of the study participants

The final versions of the Hong Kong EBP-S measures, produced by Stage VI, were included in the survey. We invited a total of 368 students: 152 BSc-Y3, 137 BSc-Y4, 48 MPT-Y2, and 31 MSc-Y2. Of the 368, 335 students responded to the survey (response rate = 91%); BSc-Y3 (n = 138), BSc-Y4 (n = 123), MPT-Y2 (n = 45), and MSc-Y2 (n = 29). Table 1 presents the baseline participant characteristics. For the retest survey, 50 students provided responses. The second survey was completed over a period of 17 days, with a range of 14 to 20 days. The S3 Table contains the follow-up subsample characteristics.

**Table 1:** Characteristics of the baseline study participants (n = 335).

Characteristics	BSc–Y3	BSc–Y4	MPT-Y2	MSc-Y2	All
**N** (%)	138 (41.2)	123 (36.7)	45 (13.4)	29 (8.7)	335 (100)
**Gender**, n (%)					
Male	78 (56.5)	70 (56.9)	18 (40.0)	21 (72.4)	187 (55.8)
Female	60 (43.5)	53 (43.1)	27 (60.0)	8 (27.6)	148 (44.2)
**Age (years)**					
mean (SD)	19.9 (0.6)	20.7 (0.6)	27.8 (4.0)	28.3 (3.8)	22 (3.7)
**GPA**, n (%)					
2.3–3.0	18 (13.0)	9 (7.3)	14 (31.1)	1 (3.5)	42 (12.5)
3.0–3.3	54 (39.1)	41 (33.3)	10 (22.2)	4 (13.8)	109 (32.5)
3.4–3.7	25 (18.1)	18 (14.6)	4 (8.9)	8 (27.6)	55 (16.4)
3.8–4.0	2 (1.5)	1 (0.8)	0 (0.0)	0 (0.0)	3 (0.9)
Prefer not to say	38 (27.5)	54 (43.9)	17 (38.8)	16 (55.2)	125 (37.3)
Missing	1 (0.7)				1 (0.3)
**Prior research experience#**, n (%)					
No	127 (92.0)	117 (95.1)	34 (75.6)	18 (62.1)	296 (88.4)
Yes	11 (8.0)	6 (7.3)	11 (24.4)	9 (31.0)	37 (11.0)
Missing				2 (6.9)	2 (0.6)

SD: Standard Deviation; GPA: Grade Point Average; BSc–Y3: Bachelor of Science–year 3; BSc–Y4: Bachelor of Science–year 4; MPT-Y2: Masters in physical therapy; MSc–Y2: Master of Science in manipulative therapy–year 2; #: Prior research experience was defined by either participating in a capstone project during a prior BSc or by working previously as a research assistant.

#### Use of EBP

Students indicated that they needed to use the EBP steps multiple times across different course subjects because many subjects require students to do seminar presentations. This consists of conducting a literature search and applying the findings to case studies or patient scenarios. During Stage 1, students suggested that to give more meaningful scoring, a rescore to the response options for each item should be applied as follows: “No Use” for the “Never” option; “Minimal Use” for “1 to 2 times”; “Regular Use” for both “Almost every month” and “2 to 10 times a month”; and “High Use” for “More than 10 times a month”. Table 2 presents the results for the “use of EBP” measure. The new total score ranges from 0 to 27 and is calculated by multiplying the response options for each item by the number of items. To facilitate the interpretation of the new total score, we created cut-off points by averaging the distance between the minimum and maximum cross-products of response options and number of items, similar to our previous approach [47]. Then, we interpreted the distance between these midpoints (S2 File). The total score for the "use of EBP" measure can therefore be described as follows: "No Use" for a total score range of (0 to 4.5)/27, "Minimal Use" for (4.6 to 13.5)/27, "Regular Use" for (13.6 to 22.5)/27, and "High Use" for (22.6 to 27)/27.

**Table 2:** Results of analysis for the “use of EBP” measure.

*Instructions*: *In the past 6 months*, *how often have you________*						
Item	Description of Item:	Never	1 to 2 times	Almost every month	2 to 10 times a month	More than 10 times a month
1	Identified a gap in your knowledge related to a patient or case situation (e.g., history, assessment, treatment)?	No Use	Minimal Use	Regular Use		High Use
2	Formulated a question to guide a literature search based on a gap in your knowledge?	No Use	Minimal Use	Regular Use		High Use
3	Effectively conducted an online literature search to address the question?	No Use	Minimal Use	Regular Use		High Use
4	Critically appraised the strengths and weaknesses of research methods (e.g., appropriateness of study design, recruitment, data collection and analysis)?	No Use	Minimal Use	Regular Use		High Use
5	Critically appraised the measurement properties (e.g., reliability and validity, sensitivity and specificity) of standardized tests or assessment tools you are considering using in your practice?	No Use	Minimal Use	Regular Use		High Use
6	Interpreted study results with the use of statistical tests and procedures (e.g., t-tests, logistic regression?)	No Use	Minimal Use	Regular Use		High Use
7	Determined if evidence from the research literature applies to your case study’s situation?	No Use	Minimal Use	Regular Use		High Use
8	Determined on an appropriate course of action based on integrating the research evidence, clinical judgment and patient or client preferences?	No Use	Minimal Use	Regular Use		High Use
9	Continually evaluated the effect of your course of action on your patient’s/client’s outcomes?	No Use	Minimal Use	Regular Use		High Use

#### EBP activities

For this measure, we did not alter the scoring of the original measure, including its total score. Students who participated in the pilot study (Phase I) concurred with the response options provided for the items, as some students engaged in the activities listed in this measure daily, weekly, biweekly, or monthly. Consequently, the committee approved the student feedback with no modifications. Table 3 presents the results of the EBP activities measure.

**Table 3:** Results of analysis for “EBP activities” measure.

*Instructions*: *In your clinical placement*, *how often have you*____________						
Item	Description of Item	Never	Monthly or less	Bi-weekly	Weekly	Daily
1	Integrated research evidence with your expertise?	0	1	2	4	20
2	Informally (e.g., chatting) shared and discussed literature/research findings with colleagues at your educational organization?	0	1	2	4	20
3	Formally (e.g. during team meetings) shared and discussed literature/research findings with colleagues at your educational organization?	0	1	2	4	20
4	Shared and discussed literature/research findings with patients/clients?	0	1	2	4	20
5	Read published research reports?	0	1	2	4	20
6	Allocated time to read research?	0	1	2	4	20
7	Attended in-services/workshops/courses in your organization about EBP?	0	1	2	4	20

#### Knowledge about EBP

**Fig 2:**
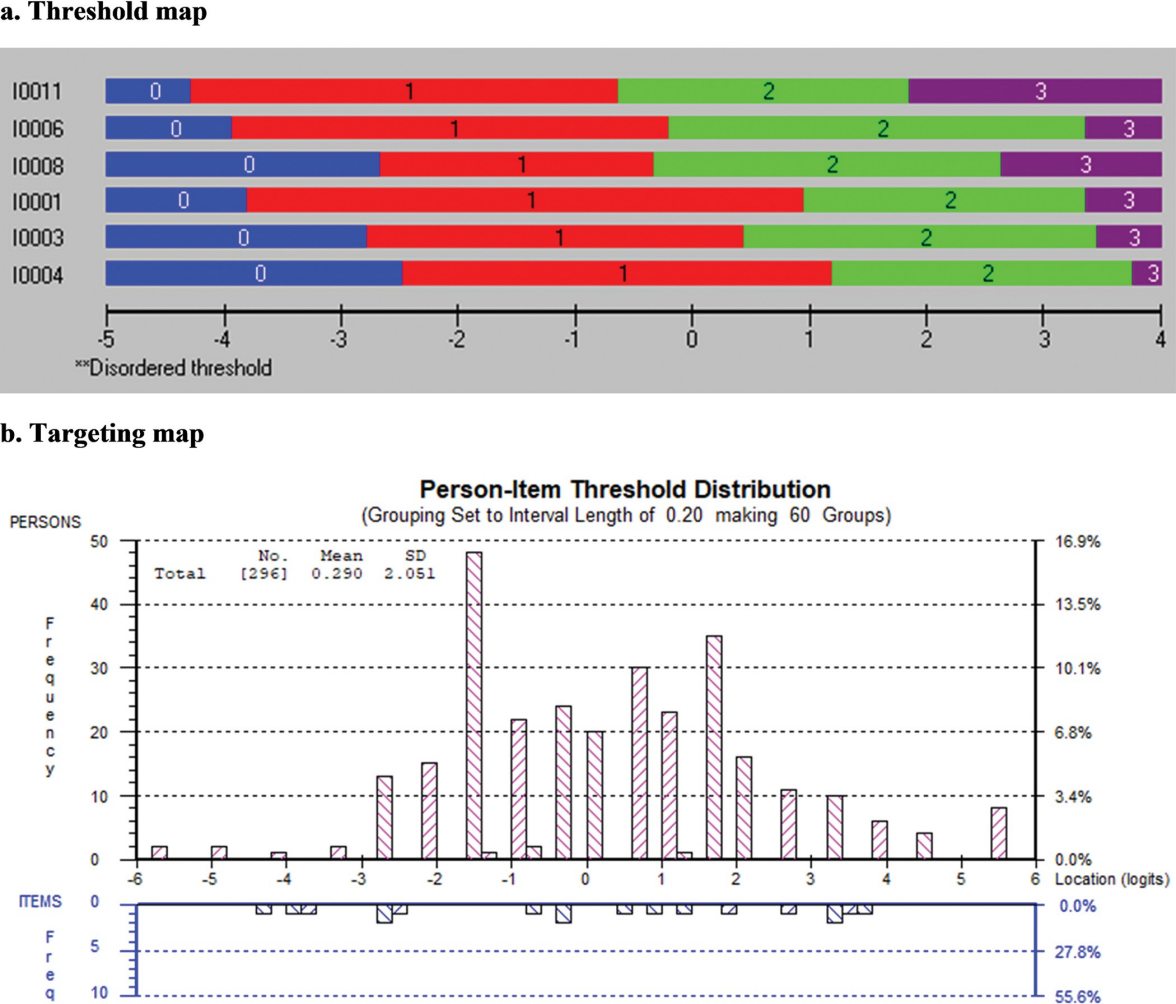
a Threshold map and b targeting map of “Knowledge about EBP” construct.

Table 4 presents the results of the Rasch analysis for the original 11 items on knowledge about EBP. All items had disordered thresholds and were rescored by collapsing the categories "never heard the term" and "have heard it but don’t understand". After rescoring, five items were removed because of a misfit with the Rasch model. One item showed dependency on another (items 1, 2). We kept the item with the best wording and removed item 2. There were no DIF by academic cohort, prior research experience, GPA, or sex. The remaining six items formed the knowledge measure for the students (*χ*^2^ = 34.46, df = 24, *p* = 0.08). Fig 2A illustrates the threshold map for the final 6-item knowledge measure, displaying the items from the simplest (randomized controlled trial) to the most difficult (meta-analysis). Fig 2B depicts the targeting map, which presents how participants were targeted by the items. This figure demonstrates that the students were reasonably well targeted by the final six items, with a mean person location of 0.29 (expected 0) and a standard deviation (SD) of 2.05 (expected 1).

**Table 4:** Results of Rasch analysis for the original items of the “Knowledge about EBP” measure.

*Instructions*: *Please indicate your level of agreement with the following statements*: *5-point Likert Scale*					
Item	Description of Item	Response option rescored	Results		
			Item misfit	Local item dependency	DIF
1	Reliability of outcome measures	√	No	Yes	No
2	Validity of outcome measures	√	No	Yes with items 1 (Item 2 deleted)	No
3	Sensitivity/Specificity of outcome measures	√	No	No	No
4	Meta-analysis	√	No	No	No
5	Confidence Interval	√	Yes (deleted)	No	No
6	Systematic Review	√	No	No	No
7	Number needed to treat	√	Yes (deleted)	No	No
8	Statistical significance	√	No	No	No
9	Minimally important change (MIC)	√	Yes (deleted)	No	No
10	Treatment effect size	√	Yes (deleted)	No	No
11	Randomized controlled trial (RCT)	√	No	No	No

#### Self-efficacy towards EBP

**Fig 3:**
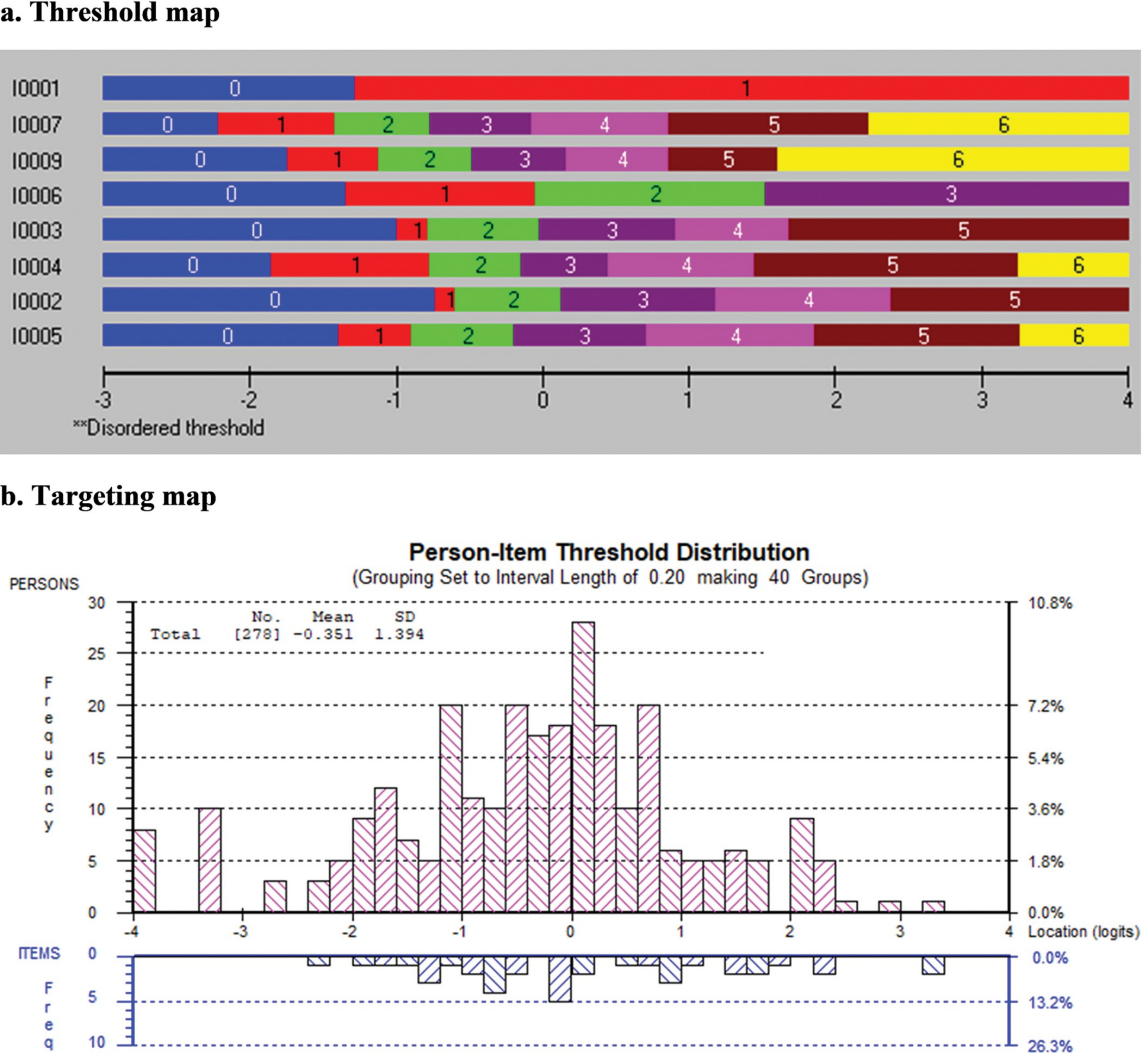
a Threshold map and b targeting map of “Self-efficacy toward EBP” construct.

Table 5 presents the results of the Rasch analysis for self-efficacy towards EBP. All items were rescored because of disordered thresholds. The lower categories ("0%", "10%", and "20%") were collapsed for five entries (items 4, 5, 6, 7, 8, and 9). For two items, the categories ("0%", "10%", "20%", and "30%") were collapsed (items 2, 3). A severely disordered item was rescored using binary categories (item 1). Item 6 was further rescored to merge the middle ("30%" with "40%" and "50%" with "60%") and higher ("70%", "80%", "90%", and "100%") categories. For six items, the upper three categories ("80%", "90%", and "100%") were merged together (items 2, 3, 4, 5, 7, and 9). All nine items fit the Rasch model. Item 8 showed dependency with item 9, and as the best-worded item was retained, item 8 was removed. There was no DIF for any of the items. The remaining eight items formed the knowledge measure for the students (χ^2^ = 25.11, df = 32, p = 0.80). The threshold map for the final self-efficacy measure is shown in Fig 3A, with item 1 being the simplest and item 5 being the most difficult. Fig 3B presents the targeting map, which shows a reasonably well targeting of the sample by the remaining 8 items, with the person mean location (SD) = -0.35 (1.39).

**Table 5:** Results of Rasch analysis for the original items of the “Self-efficacy towards EBP” measure.

*Instructions*: *Please indicate your level of agreement with the following statements*: *5-point Likert Scale*					
Item	Description of Item	Response option rescored	Results		
			Item misfit	Local item dependency	DIF
1	Identify a gap in your knowledge related to a patient or client situation (e.g., history, assessment, treatment)?	√	No	No	No
2	Formulate a question to guide a literature search based on a gap in your knowledge?	√	No	No	No
3	Effectively conduct an online literature search to address the question?	√	No	No	No
4	Critically appraise the strengths and weaknesses of study methods (e.g., appropriateness of study design, recruitment, data collection and analysis)?	√	No	No	No
5	Critically appraise the measurement properties (e.g., reliability and validity, sensitivity and specificity) of standardized tests or assessment tools that you are considering using in your practice?	√	No	No	No
6	Interpret study results obtained using statistical tests and procedures (e.g., t-tests, logistic regression)?	√	No	No	No
7	Determine if evidence from the research literature applies to your patient’s/client’s situation?	√	No	No	No
8	Decide on an appropriate course of action based on integrating the research evidence, clinical judgment and patient or client preferences?	√	No	Yes with item 9 (item 8 deleted)	No
9	Continually evaluate the effect of your course of action on your patient’s/client’s outcomes?	√	No	Yes with item 8 (item 8 deleted)	No

#### Attitudes towards EBP

**Fig 4:**
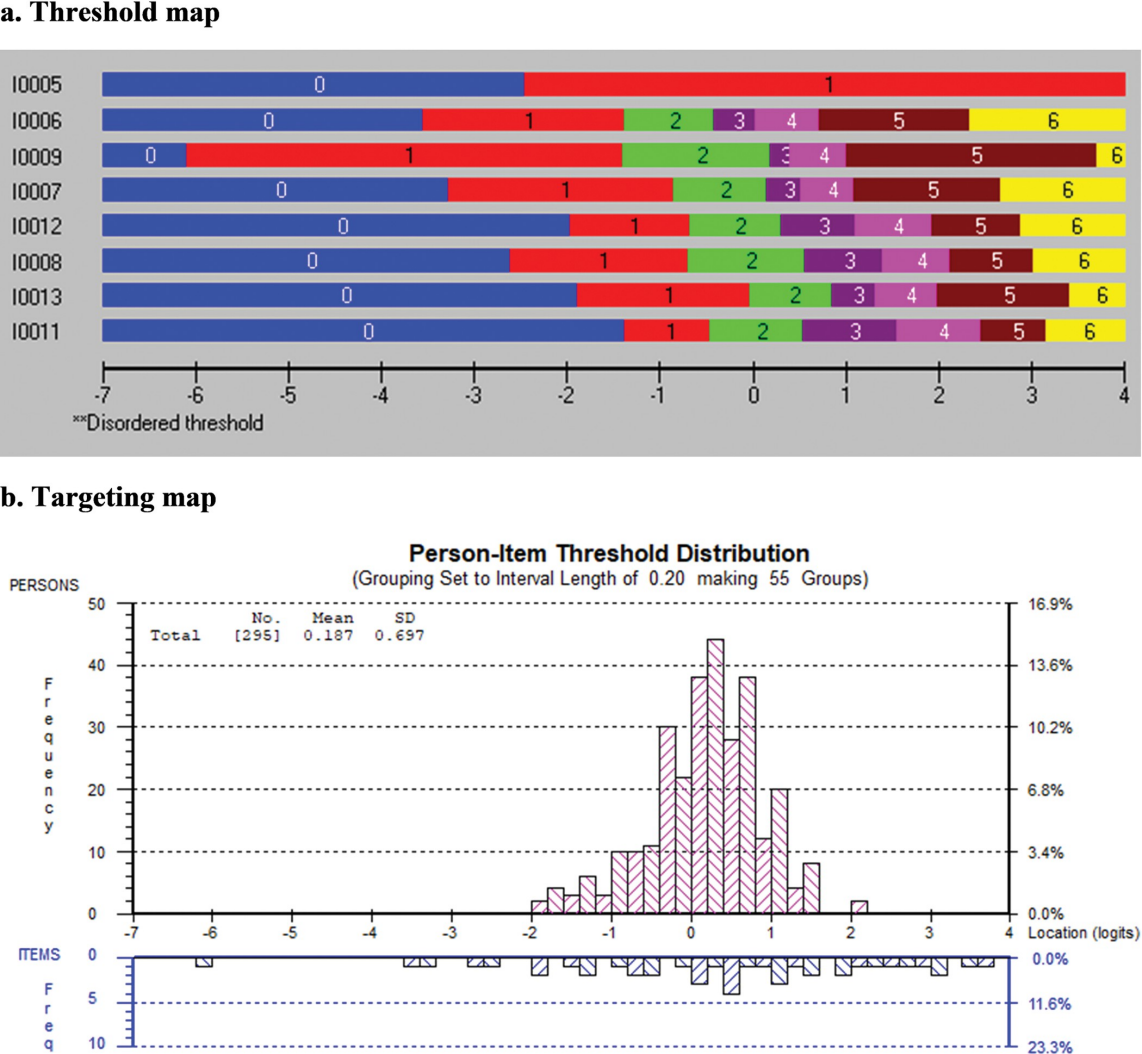
a Threshold map and b targeting map of “attitudes towards EBP” construct.

The findings of the Rasch analysis for attitudes towards EBP are shown in Table 6. Five severely disordered items were rescored by collapsing their categories into binary items (items 1, 2, 3, 4, and 5). The lower and middle categories ("Strongly disagree", “Disagree", "Somewhat disagree", and "Neither disagree nor agree") were collapsed as 0, while the upper categories ("Somewhat agree", "Agree", and "Strongly agree") were merged as 1. The remaining 8 items had negative meaning; therefore, we rescored them by reversing the order of the category options. One item misfit the Rasch model, thus it was removed (item 10). However, five items demonstrated interdependence (items 1, 2, 3, 4, and 5). The item with the best wording was retained, resulting in the removal of items 1, 2, 3, and 4. The remaining 8 items formed the attitudes towards EBP measure for the students; nonetheless, the global fit was poor (*χ*^2^ = 61.49, df = 32, *p* = 0.00). Fig 4A shows the final gradient distribution of the attitudes towards EBP items, from the least to the most difficult. Fig 4B shows a reasonably well targeting of the sample by the remaining 8 items, with the person mean location (SD) = 0.19 (0.70). It also shows more items at the lower and upper ends of the attitude’s continuum with no students.

**Table 6:** Results of Rasch analysis for the original items of the “Attitudes towards EBP” measure.

*Instructions*: *Please indicate your level of agreement with the following statements*: *5-point Likert Scale*					
Item	Description of Item	Response option rescored	Results		
			Item misfit	Local item dependency	DIF
1	EBP is considered an essential element of my clinical practice	√	No	Yes with Item 5 (Item 1 deleted)	No
2	EBP adds credibility to my profession	√	No	Yes with Item 5 (Item 2 deleted)	No
3	Using EBP improves the quality of care delivered to clients	√	No	Yes with Item 5 (Item 3 deleted)	No
4	Using EBP helps a clinician stay informed about new treatment interventions	√	No	Yes with Item 5 (Item 4 deleted)	No
5	It is important for clinicians to keep up-to-date with research evidence	√	No	Yes	No
6	EBP ignores the client’s preferences**	√	No	No	No
7	Research findings are presented in a language that is difficult to understand**	√	No	No	No
8	Applications to clinical practice are not always outlined in research studies**	√	No	No	No
9	EBP is a cook-book approach to therapy**	√	No	No	No
10	EBP disregards clinical experience**	√	Yes	No	No
11	Clinical experience is more important than research evidence when making clinical decisions **	√	No	No	No
12	EBP takes too much time**	√	No	No	No
13	EBP is only possible when the appropriate resources are available**	√	No	No	No

**Items response options were reversed as the meaning is negative.

#### Perception of the teaching and assessment of EBP in the curriculum

Table 7 presents the results of the Rasch analysis for the perception of the teaching and assessment of EBP in the curriculum. The response options in the lower categories for 20 items were disordered. Nine items were rescored by combining the lower categories "Strongly disagree" and "Disagree" (items 3, 6, 10, 12, 13, 20, 21, 22, and 36). Eleven items were rescored by collapsing the lower three categories ("Strongly disagree", "Disagree", and "Somewhat disagree") (items 1, 2, 4, 8, 9, 21, 23, 25, 26, 29, and 40). Four items misfit the Rasch model and were eliminated (items 6, 20, 22, and 32). Twenty-five items demonstrated dependency on one or more other items (items 1, 2, 3, 4, 5, 6, 7, 8, 9, 10, 11, 12, 14, 15, 16, 17, 18, 19, 27, 28, 30, 31, 38, 39, and 40). We kept the best-worded items and removed 14 others (items 2, 3, 5, 7, 9, 10, 12, 15, 16, 17, 18, 28, 31, and 39). Three items showed DIF: two by academic cohort (items 30 and 35) and one by prior research experience (item 33). Items 30 and 35 were split by the academic cohort to maintain their inclusion in this construct. However, the split items were highly correlated with two other items (items 33 and 34); hence, we eliminated the split items, resulting in the removal of items 30 and 35 from the construct. We split item 33 into two items: one for those who answered “Yes” for prior research experience and one for those who answered "No". However, the "Yes" split item was highly correlated with item 40; hence, it was removed due to its high dependency.

**Table 7:** Results of Rasch analysis for the original items of the “Perception of the teaching and assessment of EBP in the curriculum” measure.

*Instructions*: *Please indicate your level of agreement with the following statements*: *5-point Likert Scale*					
Item	Description of Item	Response option rescored	Results		
			Item misfit	Local item dependency	DIF
1	EBP is an integral part of the curriculum	√	No	No	No
2	It is the responsibility of the program to help me become an evidence-based clinician	√	No	Yes with Item 1 and 3 (Item 2 deleted)	No
3	EBP is integrated in all of our professional courses	√	No	Yes with Item 1, 4, and 31 (Item 3 deleted)	No
4	This program has provided me with a strong foundation in EBP	√	No	No	No
5	This program emphasizes the importance of personal judgment when it comes to implementing EBP		No	Yes with Item 4 (Item 5 deleted)	No
6	I feel comfortable asking professors to explain research findings that I do not understand	√	Yes	No	No
7	I feel comfortable asking professors to explain the clinical applications of research evidence		No	Yes with Item 6, 8, 9 (Item 7 deleted)	No
8	My teachers present the clinical applications of research evidence	√	No	No	No
9	My professors are good role models for EBP	√	No	Yes with Item 6 and 8 (Item 9 deleted)	No
10	My professors demonstrate positive attitudes towards EBP in the classroom	√	No	Yes with Item 8 (Item 10 deleted)	No
11	Guest clinical lecturers demonstrate positive attitude towards EBP in the classroom		No	No	No
12	Guest clinical lecturers are good role models for EBP	√	No	Yes with Item 3 and 11 (Item 12 deleted)	No
13	Guest clinical lecturers help me understand how to incorporate evidence into practice in today’s clinical environment or case study	√	No	No	No
14	I have received adequate training in order to formulate an answerable research question in the PICO format		No	No	No
15	I have received adequate training in order to search for scientific articles		No	Yes with Item 14, 16, 17 and 18 (Item 15 deleted)	No
16	I have received adequate training in order to critically appraise the scientific articles I find		No	Yes with Item 15, 17, 18 and 19 (Item 16 deleted)	No
17	I have received adequate training in order to understand the different levels of evidence for treatment effectiveness		No	Yes with Item 14, 15, 16, 18 and 19 (Item 17 deleted)	No
18	I have received adequate training to help me understand different kinds of scientific research designs (randomized control trail, cohort study, cross-sectional) in order to implement EBP		No	Yes with Item 14, 15, 16, 17 and 19 (Item 18 deleted)	No
19	I have received adequate training on how to apply the results of various studies to clinical case scenarios		No	No	No
20	I need more EBP training in order to apply it in practice**	√	Yes	No	No
21	My assigned course readings help me keep up-to-date with research evidence	√	No	No	No
22	I would rather learn about interventions from currently practicing clinicians than from scientific literature	√	Yes	No	No
23	Clinical case scenarios help me apply EBP in the classroom	√	No	No	No
24	My coursework (e.g. assignments, readings, papers, quizzes) helps me to understand how I can apply EBP in the clinical context	√	No	No	No
25	I am comfortable using systematic reviews to gather evidence	√	No	No	No
26	I am encouraged to use research in my class assignments	√	No	No	No
27	I receive feedback from my professors about the quality of scientific evidence I use in my assignments		No	No	No
28	Exams adequately evaluate my learning of EBP concepts		No	Yes with Item 27 (Item 28 deleted)	No
29	Assignments adequately evaluate my learning of EBP concepts	√	No	No	No
30	During my clinical placement, I had opportunities to apply the EBP knowledge and skills acquired from my academic program		No	No	By cohort (Item 30 deleted)
31	The settings in which I had my clinical placement promoted EBP		No	Yes with Item 3 and 30 (Item 28 deleted)	No
32	I did not have enough time to use EBP during my clinical placement**		Yes	No	No
33	My clinical educators were good role models for using EBP		No	No	By Prior research experience (item split, “Yes” item deleted due to dependency with item 40)
34	During my clinical placement, I had adequate time to search for evidence		No	No	
35	In my clinical placement, I was encouraged to implement EBP		No	No	By cohort (Item 35 deleted)
36	During my clinical placement, I saw the value of using EBP to guide clinical decisions	√	No	No	No
37	During my clinical placement, I had access to the required resources to search for evidence		No	No	No
38	I was sufficiently prepared to implement EBP when I began my clinical placement		No	No	No
39	During my clinical placement, I felt comfortable discussing the application of EBP with my supervisor		No	Yes with Item 38 and 40 (Item 39 deleted)	No
40	My clinical educator provided feedback on my EBP skills	√	No	No	No

**Items response options were reversed as the meaning is negative.

**Fig 5:**
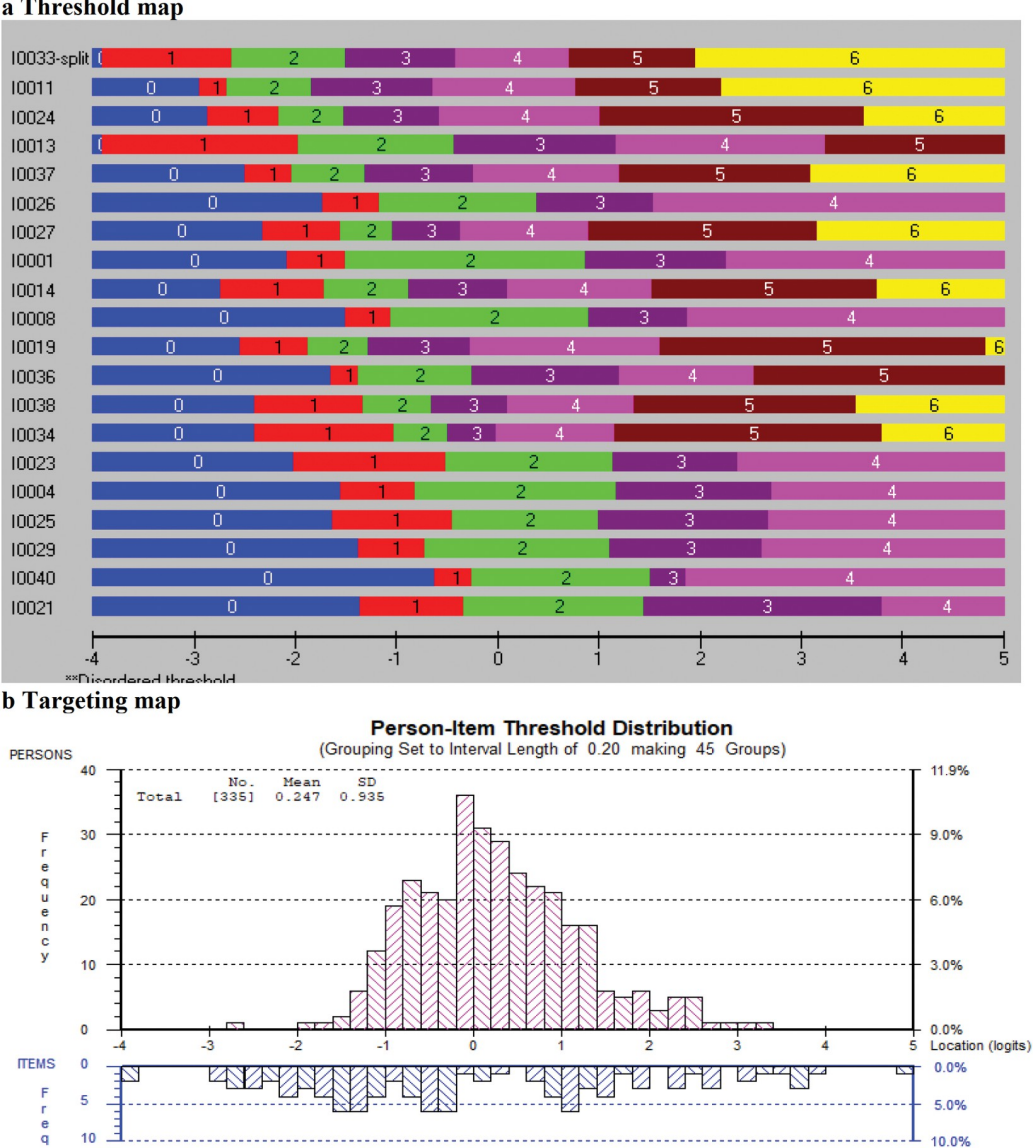
a Threshold map and b targeting map of “Perception of the teaching and assessment of EBP in the curriculum” construct.

The remaining 20 items reflecting the perception of the teaching and assessment of EBP in the curriculum fit the Rasch model and formed a measure (χ^2^ = 80.99, df = 80, *p* = 0.45). Fig 5A shows the threshold map for the final perception of the teaching and assessment of EBP in the curriculum, with the "No" split for item 33 being the easiest and item 21 being the most difficult item. Fig 5B presents the targeting map, displaying that our sample is well targeted by the 20 items, with a mean location (SD) of 0.25 (0.93).

#### Test-retest reliability

Table 8 presents the test-retest reliability results of the six EBP-S measures. The test-retest sample size was the same for all measures (n = 50), except for the “perception of the teaching and assessment of EBP in the curriculum” (n = 46). Excluding self-efficacy and attitudes, all measures on the retest showed a slight decline in scores. There was no significant test-retest mean difference across the EBP-S measures, other than knowledge, which showed an 8% change from the baseline score. The ICC_AGREEMENT_ values ranged from good for five measures (use of EBP, knowledge, self-efficacy, attitudes, and perception of the teaching and assessment of EBP in the curriculum) to excellent for one measure (EBP activities).

**Table 8:** Test retest reliability of the EBP-S measures (n = 50).

Characteristics	Use of EBP	EBP Activities	Knowledge about EBP	Self-efficacy	Attitudes towards EBP	Perception of the teaching and assessment of EBP in the curriculum
**n**	50	50	50	50	50	46
**Number of items**	9	7	6	8	8	20
**Scale range**	0–27	0–140	0–18	0–38	0–43	0–100
**Mean ± SD score 1**^**st**^ **measure**	12.82 (5.29)	20.94 (22.60)	11.2 (4.05)	17.6 (8.74)	19.6 (5.51)	58.9 (10.66)
**Mean ± SD score 2**^**nd**^ **measure**	12.68 (5.42)	20.92 (22.82)	10.3 (4.17)	18.6 (9.42)	20.0 (4.92)	55.9 (13.56)
**Mean difference absolute (95% CI)**	-0.14 (-0.76 to 1.04)	-0.02 (-1.47 to 1.51)	-0.88 (-1.66 to 0.10)	1.0 (-0.62 to 2.62)	0.38 (-0.80 to 1.56)	-2.9 (-5.8 to 0.02)
**Mean difference relative to score of 1**^**st**^ **measure**	0.7%	0.1%	8%	5.7%	2%	4.9%
**P value for mean difference**	0.76	0.98	0.03	0.22	0.52	0.05
**Missing data for the 1**^**st**^ **measure**	0%	0%	11.6%	17%	11.9%	0%
**Internal consistency** *			0.85	0.89	0.71	0.92
**ICC**_**AGREEMENT**_	0.91	0.98	0.96	0.89	0.82	0.80
**95% CI for ICC**	0.83 to 0.95	0.97 to 0.99	0.75 to 0.92	0.81 to 0.94	0.68 to 0.90	0.64 to 0.89
**P value for ICC**	< 0.01	< 0.01	< 0.01	< 0.01	< 0.01	< 0.01
**SEM**_**AGREEMENT**_	1.6	3.2	0.8	3	2.2	5.4
**SEM**_**AGREEMENT**_ **(relative to scale range)**	5.7%	2.3%	4.2%	7.7%	5%	5.5%
**MDC**	2.3	4.5	1.2	4.3	3.1	7.4
**MDC**_**90**_	3.7	7.4	1.9	7.0	5.1	12.6
**MDC**_**95**_	4.5	8.9	2.3	8.3	6.1	15.0

*Calculated using Person Separation Index (PSI)

#### Internal consistency reliability

The PSI values for knowledge, self-efficacy, attitudes, and perception of the teaching and assessment of EBP in the curriculum were 0.85, 0.89, 0.71, and 0.92, respectively (Table 8). The values of the PSI suggest acceptable internal consistency for all EBP-S measures.

#### SEM and sensitivity to change

Table 8 presents the SEM_AGREEMENT_, MDC, MDC_90_ and MDC_95_ values using the scoring method for the EBP-S measures, including the Rasched measures. The SEM_AGREEMENT_ values for all EBP-S measures ranged between 2.3% and 7.7% relative to the overall scale range. As all of these measures are less than 10%, the measurement error for these measures is satisfactory [48]. The MDC_95_ values for the EBP-S measures varied between 2.3 units for knowledge and 15 units for the perception of the teaching and assessment of EBP in the curriculum.

#### Feasibility and floor and ceiling effects

There were no missing values for three EBP-S measures in the baseline data: use of EBP, EBP activities, and perception of the teaching and assessment of EBP in the curriculum. Knowledge (11.6%), self-efficacy (17%), and attitudes (11.9%) had missing data. The use of EBP, EBP activities, attitudes towards EBP, and perception of the teaching and assessment of EBP in the curriculum exhibited neither a floor nor a ceiling impact. Self-efficacy had some floor impact, although it was 15%. Knowledge about EBP demonstrated both floor and ceiling effects; however, the values were less than 15%.

Table 9 summarizes the results of the Rasch analyses for the EBP-S measures.

**Table 9:** Summary of the Rasch analyses for the EBP-S measures.

Construct	Items		Thresholds	*N* at ceiling	p-value for global fit	PSI	Threshold range	Item location, mean (SD)	Person location, mean (SD)
	Start	Finish							
Knowledge	11	6	18	296 (88%)	0.08	0.85	-4.3 to 3.8	0.00 (0.63)	0.29 (2.05)
Self-efficacy	9	8	38	278 (83%)	0.80	0.89	-2.2 to 3.3	0.00 (0.60)	-0.35 (1.40)
Attitudes	13	8	43	295 (88%)	0.00*	0.71	-6.1 to 3.8	0.00 (1.13)	0.19 (0.70)
Perception of EBP in education	40	20	100	335 (100%)	0.45	0.92	-3.5 to 4.7	0.00 (0.46)	0.25 (0.93)
All	73	39							

EBP: Evidence-based practice; PSI: Person Separation Index; SD: Standard deviation; PSI: Person Separation Index; *All items fit the Rasch model, but improvements are still needed

## Discussion

This study culturally adapted and modified a six-domain, 89-item EBP-S measure [6, 14] originally developed for a Canadian context to a Hong Kong version for undergraduate and graduate physiotherapy students in accordance with the recommended guidelines [19]. As compared to the original study [14], two measures fit the formative model without item reduction (use of EBP (9 items) and EBP activities (7 items)) and two measures fit the reflective model with item reduction using Rasch analysis (self-efficacy (8 items) and knowledge (6 items)). For the second source of measures [6], two measures fit the reflective model with a reduction in the items using Rasch analysis (attitudes towards EBP (6 items) and perception of the teaching and assessment of EBP in the curriculum (20 items)). The resulting questionnaire demonstrated excellent psychometric properties. The findings support the use of the Hong Kong EBP-S measure in future educational programs to elicit individual and contextual factors among EBP learners.

### Use of EBP and EBP activities

The two measures underwent minimal modifications, primarily involving linguistic adjustments to align with the specific cultural and linguistic nuances of the local setting.

### Knowledge about EBP

The final six items fit the Rasch model with a mean value > 0, suggesting that the students reported a higher level of knowledge about EBP than was expected. Given that BSc-Y4, MPT-Y2, and MSc-Y2 students had conducted some research as part of their capstone projects, this finding was not surprising and is consistent with the results of the Canadian physiotherapy and occupational therapy new graduates study using the original measure [14]. This result was also consistent with previous EBP measures developed in nursing across countries using Rasch analysis [49–52]. Although these six items covered almost the entire range of the continuum (from -4 to + 4), our sample was not adequately targeted by these items. Although the original measure had eight questions [14], the knowledge items were considered to be insufficient. Future refinement of the knowledge about EBP construct should consider including additional items. Knowledge about EBP had good internal reliability, indicating that the items could adequately differentiate our students along the continuum, a finding that is comparable to the original study [14].

### Self-efficacy towards EBP

The remaining eight items fit the Rasch model, and our sample was reasonably well targeted by these items. The mean person fit of < 0 suggests a lower degree of self-efficacy for EBP than expected. This finding contradicts the results of the Canadian study using the original measure, which indicated a higher degree of self-efficacy [14]. The discrepancy may be because the original measure was administered to recent graduates who had completed their EBP training, while most learners in our sample consisted of undergraduates in their third and final year of study who likely need more time to develop confidence in their abilities to apply EBP. Indeed, there is a body literature suggests that confidence is a function of time, exposure to a construct, and an opportunity to practice [53, 54].

Self-efficacy towards EBP demonstrated good internal reliability, which is comparable to the original measure [14, 55] and other measures of self-efficacy towards EBP [56, 57].

### Attitudes towards EBP

The final 8 items on attitudes towards EBP fit the Rasch model, but our sample was inadequately targeted by the items. Our sample’s mean person fit was > 0, showing that our students’ scores about attitudes towards EBP exceeded the expected values. This is an unsurprising result given that most learners in health professions such as nurses, physiotherapists, and occupational therapists report having positive attitudes towards EBP [47, 58, 59]. This is likely the outcome of the growing emphasis on the importance of EBP in health care and the many changes in health profession education programs globally to include EBP content [4, 5]. However, multiple items in the lower (≤ -2.0 logits) and upper (≥ 2.0 logits) ends of the continuum range without any participants. Although all eight items fit the Rasch model, there is a global misfit in the construct, suggesting that the items should be further revised. This misfit may be explained by the nature of our sample; we recruited students who may have had little exposure to EBP compared to the original measure, which recruited recent graduates within 6 weeks of program completion.

The internal consistency of the attitudes towards EBP is acceptable, suggesting that the items can adequately divide students along the continuum. Our original work showed unacceptable internal reliability for the attitudes measure [14]. Our findings also contrast with a previous study that employed the Rasch model to examine the internal reliability of the attitudes measure, demonstrating a good value [60]. These differences in the internal consistency may be attributed to using Rasch with small samples for both the original measure [14] and the previous study [60] and confirm the importance of having a sufficiently large sample size in measurement work of this nature.

### Perception of the teaching and assessment of EBP in the curriculum

The final 20 items reflecting the perception of the teaching and assessment of EBP in the curriculum fit the Rasch model and targeted our sample well. The mean value for our sample was > 0, suggesting that students’ perception of their EBP education in the curriculum was more favorable than expected. The number of items available for this measure almost covers the hierarchical continuum scale. Three items showed DIF: items 30 and 35 by academic cohorts, and item 33 by prior research experience. Item 30 showed that BSc-Y3 students reported lower levels of agreement about having opportunities to apply the EBP knowledge and skills in their clinical placement. Item 35 also showed that BSc-Y3 students felt that they were less encouraged to implement EBP during their clinical placement compared to other cohorts. This is unsurprising because this cohort only had 10 days of clinical placement, which is likely insufficient to develop a clear idea about the impact of their education on EBP during their clinical placement. Moreover, our data were collected during the COVID-19 pandemic, when all face-to-face teaching and clinical placement pivoted to online formats. This transformation significantly impacted the learning process, from knowledge acquisition to application, in all Hong Kong postsecondary institutions [61]. Item 33 revealed that students who had prior research experience, either from previous capstone projects or from working as research personnel, were less likely to agree that their clinical educators were effective role models for EBP use. It appears that students with prior research experience may have differing expectations and perceptions regarding the application and modeling of EBP. Students may believe that some of their educators were successful in demonstrating EBP, while others did not meet their expectations due to a lack of knowledge, a hefty workload, or resistance to change. These challenges were also cited by clinical educators [62].

The internal consistency of this measure was excellent. A similar finding was reported by the Osteopathy Clinical Teaching Questionnaire [63], which explores the students’ perceptions of the instructors’ clinical teaching during clinical placement.

### Measurement properties

For internal consistency and measurement error, the values of PSI and SEM_AGREEMENT_ are good and acceptable for all measures. However, for test-retest reliability, it is hard to draw conclusions due to the large 95% CI. In terms of feasibility, three measures had missing data for the total scores, which ranged between 11.6% and 17.0%. However, there is no consensus regarding the acceptable amount of missing data for a measure to establish its feasibility. In statistics, for future use of the measure, missing data of ≤5% can be handled by single imputations [64]; 5–10% can be managed by listwise deletion, imputation, or likelihood-based methods [65]; and >10% can be handled by multiple imputations [66].

### Strengths and limitations

This study had several strengths. First, the sample size was adequate to test the construct validity using Rasch analysis and reliability [24–27]. Second, our study had a high participation rate, which was likely the result of providing incentives [67]. Third, this is the first study to investigate the psychometric properties of students’ perceptions about the teaching of EBP in physiotherapy curricula. This new measure is more specific to EBP than the previous measures, which evaluated the students’ perceptions of instructors and the learning process in general [68–71].

As with any study, ours has limitations. First, our sample was diverse, ranging from students with only a few days of clinical placement to graduates with several years of clinical experience. This diversity was evident in our DIF results. Second, we administered 89 items that required 15 minutes to complete, which might negatively impact the participants’ concentration in answering the questions. However, after using Rasch, the number of items was reduced to 55, which will decrease the time required to complete the questionnaire and may enhance the completion rate in the future. Third, although the Rasch analysis revealed that the items had captured a broad range of the hierarchical continuum, there are gaps in some locations, affecting the precision of the person ’s estimates within these gaps. Fourth, despite the large sample size, participation was limited to physiotherapy students from a single tertiary institution. Although three other local institutions offer physiotherapy programs in Hong Kong, these institutions were recently founded and may have distinct EBP curriculum formats. Therefore, our findings may not be generalized to these institutions. Furthermore, any modifications made to the current curriculum would necessitate replicating this study to determine the potential inclusion of additional items within these constructs, aligning them with the revised curriculum. Future studies including these institutions are warranted to establish generalizability. Fifth, the knowledge about EBP measure in our study is limited to statistical and methodological terms, a limitation identified in the original work [14]. This measure should include items that assess students’ understanding of the 5 steps of the EBP process [3] as opposed to research-related terms such as sensitivity and specificity. Therefore, we may need to develop another measure of knowledge about using EBP steps instead of knowledge of statistical and methodological terms. In reality, both measures are required. Sixth, the data were collected during the COVID-19 pandemic, which may have influenced the students’ responses given that their entire education, including EBP, was shifted to online formats [72, 73]. Last, our measures rely on self-report to collect data about EBP, which may not reflect the actual student competence in these various constructs [74–76]. While self-reported measures are a more practical option when time, resources, and logistics are limited [77], the evaluation of education on EBP may need the integration of self-reported and performance-based measures as some EBP steps, such as developing appropriate keywords for the research question and searching the literature.

### Future implications

Psychometric properties, such as the minimal important change, should be tested to help interpret the score changes over time. The knowledge measure must be expanded to encompass the five phases of the EBP process [3]. The validation of EBP-S measures will enable the identification of the major factors influencing students’ use of EBP. This will make it possible to examine the mechanism of action of these factors [47], allowing researchers to develop a theoretical and statistical model that may influence the design of interventions that can bring about changes in what and how we teach EBP [78]. This will be explored in a subsequent analysis using this large data set.

## Conclusions

This study validated EBP-S measures for physiotherapy students. The psychometric properties of each EBP-S measure (use of EBP, EBP activities, knowledge about EBP, self-efficacy, attitudes towards EBP, and perception of the teaching and assessment of EBP in the curriculum) were estimated using cross-cultural validity, structural validity, internal consistency, test-retest reliability, SEM, and MDC. The findings indicated that the EBP-S measures possessed good psychometric properties. Rasch analysis revealed that some constructs (attitudes, knowledge, and self-efficacy) have room for improvement. The internal consistency and reliability appeared to be acceptable for all constructs. These measures can be used to identify the determinants of EBP within the specific physiotherapy curriculum in Hong Kong. However, it is crucial to recognize that enhancing the students’ knowledge and skills in EBP necessitates certain modifications to the curriculum. Consequently, it becomes imperative to replicate this study in order to ascertain the potential inclusion of additional items within these constructs, aligning with the revised curriculum.

## Supporting information

S1 File The cross-cultural adaptation process. (PDF)

S2 File Creating cut-off points for the “Use of EBP” measure. (PDF)

S1 Table Changes to the original questionnaires. (PDF)

S2 Table Characteristics of the pilot study participants (n = 52). (PDF)

S3 Table Characteristics of the follow-up subsample for test retest reliability (n = 50). (PDF)
