# Supplementary material for: Refining and adapting the measurement properties of evidence-based practice measures for physiotherapy students
Source: PLoS One. 2024 Mar 7;19(3):e0298611. doi: 10.1371/journal.pone.0298611 (PMC10919638; doi:10.1371/journal.pone.0298611)
Supplement: S1 File — (PDF) [file pone.0298611.s001.pdf]

S1 File: The cross-cultural adaptation process.

The translation and cross-cultural adaptation process followed the recommended guidelines (1). Appendix 1 details the process followed by this paper. which consists of six stages:

i. *Forward translation*: Five local translators with native-level Hong Kong English and physiotherapy training conducted independent reviews of the original North American English measures. One of them was familiar with the EBP procedure and possessed a PhD, while the other four were senior undergraduates with limited knowledge of EBP. We utilized physiotherapy-trained translators because these measures will only be utilized by physiotherapy trainees. Each translator wrote a report detailing the unclear wording, challenging items, and the suggested rephrasing.
ii. *Translation synthesis*: The forward translators discussed their reports and, under the guidance of a synthesis recorder (a physiotherapy professor, FAZ), generated an initial draft. The synthesis recorder resolved translators' divergences.
iii. *Back translation*: This step was not needed because the translation was done to another form of English.
iv. *Expert committee review*: Two expert panel committee meetings were held to produce a pre-final final version of the two EBP-S measures. A 10-peron committee with diverse educational backgrounds (i.e. five forward translators, a methodologist, two English-speaking academics from North American, two English-speaking academics from Hong Kong, and a psychometrician) compared the original two measures with the forward translation, reviewed the forward translation reports, including the word/phrasal uncertainties and suggested rewording/rephrasing. The committee met twice to ensure that the semantic (i.e., multiple meaning of a particular word) and idiomatic (e.g., presence of idioms), conceptual, and experiential equivalence between the original and translated versions. The process resulted in a pre-final Hong Kong version of the EBP-S measures. We sent the pre-final draft to three Hong Kong English-speaking academics in charge of Bachelor of Science (BSc), Master Physiotherapy program (MPT), and Master of Science (MSc) programs for feedback on language. This step was important to further verify the robustness of the semantic and idiomatic equivalence used in these physiotherapy programs.
v. *Test of the pre-final version*: The pre-final Hong Kong version of the EBP-S measures was field tested in a convenient representative sample of physiotherapy students. According to the standards (1), 30 to 40 individuals is recommended. The currently enrolled undergraduate or postgraduate physiotherapy students at The Hong Kong Polytechnic University were recruited. Specifically, invitation emails were sent to the students. A research assistant with a master's degree in public health described the goal of the study and responded to any questions through phone or email once participants agreed to participate. After providing the informed written consent, the pre-final version of the measures and sociodemographic sheet were sent to participants via email or in-person by the research assistant. Participants completed the online or paper-based survey without consulting the research assistant. After completing the survey, participants underwent semi-structured telephone or in-person cognitive debriefing sessions with the research assistant who received extensive training on the cognitive debriefing interviewing approach. These interviews aimed to determine if the cross-cultural measures' content corresponds with its intended use (1). Additionally, these interviews evaluated the level of understandability, relevance, and comprehensiveness of the pre-final version to the participants. Participants were asked to rate: (1) relevance (To which extent do you think this item is relevant to this EBP measure? Extremely irrelevant to extremely relevant; and

(2) comprehension (To which extent do you think this item is understandable and comprehensible? From very difficult to understand) using a 5-point Likert scale. from 1 to 5 about:

a) Relevancy: ‘To which extent do you think this item is relevant to this EBP measure?’ [extremely irrelevant, Irrelevant, somewhat relevant, relevant, extremely relevant].
b) Comprehension: ‘To which extent do you think this item is understandable and comprehensible?’ [Very difficult to understand, difficult to understand, somewhat understandable, easy to understand, very easy to understand].

Participants were encouraged to provide additional comments and feedback regarding the items and measures. These interviews were audio recorded in order to verbatim transcribe and analyse them.

vi. *Final version and appraisal of the adaptation*: The findings were reviewed by the expert committee members to determine whether minor modifications were needed. The original developers of the EBP-S measures then evaluated and approved the final Hong Kong version.

References

1. Beaton DE, Bombardier C, Guillemin F, Ferraz MB. Guidelines for the process of cross-cultural adaptation of self-report measures. Spine. 2000;25(24):3186-91.
