# Supplementary material for: Refining and adapting the measurement properties of evidence-based practice measures for physiotherapy students
Source: PLoS One. 2024 Mar 7;19(3):e0298611. doi: 10.1371/journal.pone.0298611 (PMC10919638; doi:10.1371/journal.pone.0298611)
Supplement: S2 File — (PDF) [file pone.0298611.s002.pdf]

S2 File: Creating cut-off points for the “Use of EBP” measure.

	Never	1 to 2 times	Almost every month	2-10 times/ month	More than 10 times a month	# of items	Total score range
Original scale	0	1	2	3	4	9	0-36
New scale	0	1	2		3	9	0-27
Meaning for the individual item	No use	Minimal use	Regular use		Maximum use		
New total score= response option per item X number of items	0	9	18		27		

Mid-points between the lower and upper cross-product of the response options and number of items: 4.5 (between 0 and 9), 13.5 (between 9 and 18), 22.5 (between 18 and 27)

	No Use	Minimal Use	Regular Use	High Use
Cut-off ranges	0.0-4.5	4.6-13.5	13.6-22.5	22.6-27.0
Meaning for the total score	No Use	Minimal Use	Regular Use	High Use
