# Supplementary material for: Refining and adapting the measurement properties of evidence-based practice measures for physiotherapy students
Source: PLoS One. 2024 Mar 7;19(3):e0298611. doi: 10.1371/journal.pone.0298611 (PMC10919638; doi:10.1371/journal.pone.0298611)
Supplement: S1 Table — (PDF) [file pone.0298611.s003.pdf]

S1 Table: Changes to the original questionnaires.

~~AaBbCcDdEe~~: Original text

AaBbCcDdEe: Modified tex

Current self-reported use of Evidence-based practice

Instructions: ~~*For each of the following activities, how often have you done the following in the past 6 months?: 5-point scale*~~ *In the past 6 months, how often have you__________*

**Description of Item:**	**Never**	**1 to 2 times**	**Almost every month**	**2 to 10 times a month**	**More than 10 times a month**
Identified a gap in your knowledge related to a patient or case ~~study~~ situation (e.g., history, assessment, treatment)?	☐	☐	☐	☐	☐
Formulated a question to guide a literature search based on a gap in your knowledge?	☐	☐	☐	☐	☐
Effectively conducted an online literature search to address the question?	☐	☐	☐	☐	☐
Critically appraised the strengths and weaknesses of research methods (e.g., appropriateness of study design, recruitment, data collection and analysis)?	☐	☐	☐	☐	☐
Critically appraised the measurement properties (e.g., reliability and validity, sensitivity and specificity) of standardized tests or assessment tools you are considering using in your practice?	☐	☐	☐	☐	☐
Interpreted study results with the use of statistical tests and procedures (e.g., t-tests, logistic regression?)	☐	☐	☐	☐	☐
Determined if evidence from the research literature applies to your case study's situation?	☐	☐	☐	☐	☐
Determined on an appropriate course of action based on integrating the research evidence, clinical judgment and patient or client preferences?	☐	☐	☐	☐	☐

Continually evaluated the effect of your course of action on your patient's/client's outcomes?	☐	☐	☐	☐	☐

EBP activities

Instructions: *In ~~the past month~~ your clinical placement, how often have you~~?: 5-point Scale~~_____________*					
**Description of Item**	**Never**	**Monthly or less**	**Bi-weekly**	**Weekly**	**Daily**
Integrated research evidence with your expertise?	☐	☐	☐	☐	☐
Informally (e.g., ~~outside of formal team or family meetings~~ hallway chatting) shared and discussed literature/research findings with colleagues at ~~work~~ your educational organization?	☐	☐	☐	☐	☐
Formally (e.g. during team ~~or family~~ meetings) shared and discussed literature/research findings with colleagues at ~~work~~ your educational organization?	☐	☐	☐	☐	☐
Shared and discussed literature/research findings with patients/clients?	☐	☐	☐	☐	☐
Read published research reports?	☐	☐	☐	☐	☐
~~Made~~ Allocated time to read research?	☐	☐	☐	☐	☐
Attended in-services/workshops/courses in your organization?	☐	☐	☐	☐	☐

Knowledge of EBP

Instructions: *Please rate your understanding of the following terms~~: 5-point Likert Scale~~*					
**Description of Item**	**Never heard the term**	**Have heard it but don't understand**	**Have some understanding**	**Understand quite well**	**Understand and could explain to others**
Reliability of outcome measures	☐	☐	☐	☐	☐
Validity of outcome measures	☐	☐	☐	☐	☐
Sensitivity/Specificity of outcome measures	☐	☐	☐	☐	☐
Meta-analysis	☐	☐	☐	☐	☐
Confidence Interval	☐	☐	☐	☐	☐
Systematic Review	☐	☐	☐	☐	☐
Number needed to treat	☐	☐	☐	☐	☐
Statistical significance	☐	☐	☐	☐	☐
Minimally important change (MIC)	☐	☐	☐	☐	☐
Treatment effect size	☐	☐	☐	☐	☐
Randomized controlled trial (RCT)	☐	☐	☐	☐	☐

Self-efficacy towards EBP

Instructions: *Please indicate how confident you are in your current level of ability by choosing the corresponding number on the following rating scale~~: 11-point Continuous Scale~~*

Description of Item	0% (No confidence)	10%	20%	30%	40%	50%	60%	70%	80%	90%	100% (Completely confident)
Identify a gap in your knowledge related to a patient or client situation (e.g., history, assessment, treatment)?	☐	☐	☐	☐	☐	☐	☐	☐	☐	☐	☐
Formulate a question to guide a literature search based on a gap in your knowledge?	☐	☐	☐	☐	☐	☐	☐	☐	☐	☐	☐
Effectively conduct an online literature search to address the question?	☐	☐	☐	☐	☐	☐	☐	☐	☐	☐	☐
Critically appraise the strengths and weaknesses of study methods (e.g., appropriateness of study design, recruitment, data collection and analysis)?	☐	☐	☐	☐	☐	☐	☐	☐	☐	☐	☐
Critically appraise the measurement properties (e.g., reliability and validity, sensitivity and specificity) of standardized tests or assessment tools that you are considering using in your practice?	☐	☐	☐	☐	☐	☐	☐	☐	☐	☐	☐

Interpret study results obtained using statistical tests and procedures (e.g., t-tests, logistic regression)?	☐	☐	☐	☐	☐	☐	☐	☐	☐	☐	☐
Determine if evidence from the research literature applies to your patient's/client's situation?	☐	☐	☐	☐	☐	☐	☐	☐	☐	☐	☐
Decide on an appropriate course of action based on integrating the research evidence, clinical judgment and patient or client preferences?	☐	☐	☐	☐	☐	☐	☐	☐	☐	☐	☐
Continually evaluate the effect of your course of action on your patient's/client's outcomes?	☐	☐	☐	☐	☐	☐	☐	☐	☐	☐	☐

Attitudes towards EBP

***Instructions**: Please indicate ~~to what extent you agree or disagree with each of~~ your level of agreement with the following statements~~?~~: 5-point Likert Scale*								
**Description of Item**	**Strongly Disagree**	**Somewhat Disagree**	**Disagree**	**Neither Agree nor Disagree**	**Somewhat Agree**	**Agree**	**Strongly agree**	~~**I don't know**~~
EBP is considered an essential element of ~~OT~~ my clinical practice	☐	☐	☐	☐	☐	☐	☐	~~☐~~
EBP adds credibility to ~~the profession of OT~~ my profession	☐	☐	☐	☐	☐	☐	☐	~~☐~~
Using EBP improves the quality of care delivered to clients	☐	☐	☐	☐	☐	☐	☐	~~☐~~
Using EBP helps ~~therapists~~ a clinician stay informed about new ~~OT~~ treatment interventions	☐	☐	☐	☐	☐	☐	☐	~~☐~~
It is important for ~~OT~~ clinicians to keep up-to-date with research evidence	☐	☐	☐	☐	☐	☐	☐	~~☐~~
EBP ignores the client's ~~wishes~~ preferences	☐	☐	☐	☐	☐	☐	☐	~~☐~~
Research findings are presented in a language that is difficult to understand	☐	☐	☐	☐	☐	☐	☐	~~☐~~
Applications to clinical practice are not always outlined in research studies	☐	☐	☐	☐	☐	☐	☐	~~☐~~

EBP is a ~~“cookie-cutter”~~ cook-book approach to therapy	☐	☐	☐	☐	☐	☐	☐	~~☐~~
EBP disregards clinical experience	☐	☐	☐	☐	☐	☐	☐	~~☐~~
Clinical experience is more important than research evidence when making clinical decisions	☐	☐	☐	☐	☐	☐	☐	~~☐~~
EBP takes too much time	☐	☐	☐	☐	☐	☐	☐	~~☐~~
EBP is only possible when the appropriate resources are available	☐	☐	☐	☐	☐	☐	☐	~~☐~~

Overall Perception of EBP in the Curriculum

	Instructions: Please indicate to what extent you agree or disagree with each of the following statements?								
**Item**	**Description of Item**	Strongly Disagree	Disagree	Somewhat Disagree	Neither Agree nor Disagree	Somewhat Agree	Agree	Strongly Agree	~~I don't know~~
1	EBP is an integral part of the ~~OT~~ curriculum	1	2	3	4	5	6	7	~~0~~
2	It is the responsibility of the ~~OT~~ program to help me become an evidence-based ~~practitioner~~ clinician	1	2	3	4	5	6	7	~~0~~
3	EBP is integrated in all of our professional courses	1	2	3	4	5	6	7	~~0~~
4	This program has provided me with a strong foundation in EBP	1	2	3	4	5	6	7	~~0~~
5	This program emphasizes the importance of personal judgment when it comes to implementing EBP	1	2	3	4	5	6	7	~~0~~
6	I feel comfortable asking professors to explain research findings that I do not understand	1	2	3	4	5	6	7	~~0~~
7	I feel comfortable asking professors to explain the clinical applications of research evidence	1	2	3	4	5	6	7	~~0~~
8	My ~~professors~~ teachers present the clinical applications of research evidence	1	2	3	4	5	6	7	~~0~~
9	My professors are good role models for EBP	1	2	3	4	5	6	7	~~0~~

10	My professors demonstrate positive attitudes towards EBP in the classroom	1	2	3	4	5	6	7	~~0~~
11	Guest clinical lecturers ~~incorporate research evidence into their teaching~~ demonstrate positive attitude towards EBP in the classroom	1	2	3	4	5	6	7	~~0~~
12	Guest clinical lecturers are good role models for EBP	1	2	3	4	5	6	7	~~0~~
13	Guest clinical lecturers help me understand how to incorporate evidence into practice in today's clinical environment or case study	1	2	3	4	5	6	7	~~0~~
14	I have received adequate training in order to formulate an answerable research question in the PICO format	1	2	3	4	5	6	7	~~0~~
15	I have received adequate ~~instruction~~ training in order to search for scientific articles	1	2	3	4	5	6	7	~~0~~
16	I have received adequate training in order to critically appraise the scientific articles I find	1	2	3	4	5	6	7	~~0~~
17	I have received adequate training in order to understand the different levels of evidence for treatment effectiveness	1	2	3	4	5	6	7	~~0~~
18	I have received adequate training to help me understand different kinds of scientific research designs (randomized control trail, cohort study, cross-sectional) in order to implement EBP	1	2	3	4	5	6	7	~~0~~

19	I have received adequate training on how to apply the results of various ~~scientific research articles/study designs~~ studies to clinical case scenarios	1	2	3	4	5	6	7	~~0~~
20	I need more EBP ~~instruction~~ training in order to apply it in practice	1	2	3	4	5	6	7	~~0~~
21	My assigned course readings help me keep up-to-date with research evidence	1	2	3	4	5	6	7	~~0~~
22	I would rather learn about interventions from currently practicing clinicians than from scientific literature	1	2	3	4	5	6	7	~~0~~
23	Clinical case scenarios help me apply EBP in the classroom	1	2	3	4	5	6	7	~~0~~
24	My coursework (e.g. assignments, readings, papers, quizzes) helps me to understand how I can apply EBP in the clinical context	1	2	3	4	5	6	7	~~0~~
25	I am comfortable using systematic reviews to gather evidence	1	2	3	4	5	6	7	~~0~~
26	I am encouraged to use research in my class assignments	1	2	3	4	5	6	7	~~0~~
27	I receive feedback from my professors about the quality of scientific evidence I use in my assignments	1	2	3	4	5	6	7	~~0~~
28	Exams adequately evaluate my learning of EBP concepts	1	2	3	4	5	6	7	~~0~~

29	~~Class~~ Assignments ~~(i.e. CBAs, midterm papers, etc)~~ adequately evaluate my learning of EBP concepts	1	2	3	4	5	6	7	~~0~~
30	During my ~~fieldwork experiences~~ clinical placement I had opportunities to apply the EBP knowledge and skills acquired from my academic program	1	2	3	4	5	6	7	~~0~~
31	The settings in which I had my ~~fieldwork experiences~~ clinical placement promoted EBP	1	2	3	4	5	6	7	~~0~~
32	I did not have enough time to use EBP during my ~~fieldwork experiences~~ clinical placement	1	2	3	4	5	6	7	~~0~~
33	My clinical educators were good role models for using EBP	1	2	3	4	5	6	7	~~0~~
34	During my ~~fieldwork experiences~~ clinical placement, I had adequate time to search for evidence	1	2	3	4	5	6	7	~~0~~
35	In my ~~fieldwork experiences~~ clinical placement, I was encouraged to implement EBP	1	2	3	4	5	6	7	~~0~~
36	During my ~~fieldwork experiences~~ clinical placement, I saw the value of using EBP to guide clinical decisions	1	2	3	4	5	6	7	~~0~~
37	During my ~~fieldwork experiences~~ clinical placement, I had access to the required resources to search for evidence	1	2	3	4	5	6	7	~~0~~

38	I was sufficiently prepared to implement EBP when I began my ~~fieldwork experiences~~ clinical placement	1	2	3	4	5	6	7	~~0~~
39	During my ~~fieldwork experiences~~ clinical placement, I felt comfortable discussing the application of EBP with my supervisor	1	2	3	4	5	6	7	~~0~~
40	My ~~supervisor~~ clinical educator provided feedback on my EBP skills	1	2	3	4	5	6	7	~~0~~
