# Supplementary material for: Refining and adapting the measurement properties of evidence-based practice measures for physiotherapy students
Source: PLoS One. 2024 Mar 7;19(3):e0298611. doi: 10.1371/journal.pone.0298611 (PMC10919638; doi:10.1371/journal.pone.0298611)
Supplement: S2 Table — (PDF) [file pone.0298611.s004.pdf]

S2 Table: Characteristics of the pilot study participants (n=52)

Characteristics	N (%)
**Gender**, n (%)	
Male	29 (55.8)
Female	23 (44.2)
**Age (years)**	
mean (SD)	21.7 (2.0)
**GPA**, n (%)	
2.3-3.0	7 (13.5)
3.0-3.3	16 (30.8)
3.4-3.7	11 (21.2)
3.8-4.0	0 (0.0)
Prefer not to say	17 (36.7)
Missing	1 (2.0)
**Current academic year**, n (%)	
BSc – Year 3	39 (75.0)
BSc – Year 4 or above	7 (13.5)
MSc – Year 2	6 (11.5)
**Prior research experience**, n (%)	
No	46 (88.5)
Yes	6 (11.5)

SD: Standard Deviation; GPA: Grade Point Average; BSc – Y3: Bachelor of Science – year 3; BSc – Y4: Bachelor of Science – year 4; MPT-Y2: Masters in physical therapy; MSc – Y2: Master of Science in manipulative therapy – year 2
