# Supplementary material for: Refining and adapting the measurement properties of evidence-based practice measures for physiotherapy students
Source: PLoS One. 2024 Mar 7;19(3):e0298611. doi: 10.1371/journal.pone.0298611 (PMC10919638; doi:10.1371/journal.pone.0298611)
Supplement: S3 Table — (PDF) [file pone.0298611.s005.pdf]

S3 Table: Characteristics of the follow-up subsample for test retest reliability (n=50).

Characteristics	N (%)
**Gender**, n (%)	
Male	29 (55.8)
Female	23 (44.2)
**Age (years)**	
mean (SD)	22 (2.0)
**GPA**, n (%)	
2.3-3.0	4 (8)
3.0-3.3	22 (44)
3.4-3.7	8 (16)
3.8-4.0	1 (2)
Prefer not to say	11 (22)
Missing	4 (8)
**Current academic year**, n (%)	
BSc – Year 3	25 (50)
BSc – Year 4 or above	19 (38)
MSc – Year 2	6 (12)
**Prior research experience**, n (%)	
No	46 (88.5)
Yes	6 (11.5)

SD: Standard Deviation; GPA: Grade Point Average; BSc – Y3: Bachelor of Science – year 3; BSc – Y4: Bachelor of Science – year 4; MPT-Y2: Masters in physical therapy; MSc – Y2: Master of Science in manipulative therapy – year 2
